# Diagnosis of pulmonary alveolar microlithiasis

**DOI:** 10.1590/0100-3984.2015.48.5e3

**Published:** 2015

**Authors:** Marcel Koenigkam Santos

**Affiliations:** 1 Attending Physician at Hospital das Clínicas – Faculdade de Medicina de Ribeirão Preto, Universidade de São Paulo (HCFMRP-USP), MD, Radiologist at MED – Medicina Diagnóstica, Ribeirão Preto, SP, Brazil. E-mail: marcelk46@yahoo.com.br.

In previous issue of **Radiologia Brasileira**, readers can find a very interesting article published by Francisco et al.^(1)^ about pulmonary alveolar microlithiasis (PAM). The authors describe the high-resolution computed tomography (HRCT) findings in chest exams of 13 patients with PAM, independently evaluated by two observers. According to the authors, diagnoses of PAM can be made on basis of clinical data compatible with the typical imaging findings, with no need for confirmation by biopsy. Thus, the mentioned article is quite interesting and current as, besides describing findings in an optimum sample of patients with a quite rare disease, utilizing a scientific methodological process recommended for this type of study, the authors highlight the increasing relevance of our specialty in recent years, as imaging findings associated with clinical data are sufficient to formulate a diagnosis, with no need for interventional procedures.

PAM is a very rarely observed disease, and the few studies describing its radiological findings are restricted to case reports or case series with a small number of patients^(2-4)^. The study of 13 well documented cases certainly adds information about tomographic assessment of this disease. For the purpose of comparison, we can mention, for example, the study developed by Deniz et al.^(5)^, published in an important European radiology journal, in which the authors evaluated 10 patients with PAM and described imaging findings at HRCT.

The methodological process is another positive point to be considered. Even considering that it is a case review article, it is necessary to highlight the relevance of an appropriate scientific method and of the correct way to present and discuss results in a text written for publication in a medical journal. In this article by Francisco et al., two experienced observers have independently evaluated the images with later consensual decision in cases of disagreement. Such analysis carries a lower chance of biases and is more reliable and reproducible as compared, for example, with a review undertaken by a single observer. There are texts from different societies offering guidance not only regarding methods applied to research in radiology, but also regarding results presentation techniques, writing of scientific texts and education in research^(6,7)^. Scientific research in the field of radiology has developed a lot in recent years, following the trend of changes and modernization of scientific methods in other areas of medicine. Such modernization includes development of the evidence-based radiology and prospective clinical trials using imaging methods^(8,9)^. Radiology, that previously rubbed shoulders with pathology, currently plays a role that goes far beyond diagnosis, and imaging findings correlation and is done with clinical data, functional status and prognosis, so that image became a biomarker^(10)^. In the case of chest imaging studies, it is also very important to use a correct terminology as proposed by both international and Brazilian documents ^(11-14)^ which were appropriately utilized in the mentioned article by Francisco et al. A correct use of terminology allows not only for an appropriate understanding of the findings, but also for exchanging accurate information between medical specialties and appropriate studies comparison.

Finally, I would like to highlight a very important aspect also described in the article by Francisco et al., which is also part of the daily routine in radiological clinics: the increased importance of the role played by radiologists. Our specialty has evolved a lot in the last years, and imaging methods accuracy showed an exponential increase. Indeed, we have increasingly made diagnoses, and definite diagnoses, without need for the well known "pathological confirmation". In chest radiology, we have an excellent example present at the most recent ATS/ERS consensus about idiopathic interstitial pneumonias^(15)^. According to the authors of this document, patients presenting with a pattern of usual interstitial pneumonia at HRCT and compatible clinical data, do not require diagnostic confirmation of idiopathic pulmonary fibrosis by biopsy. It is necessary that the radiologist assumes his role as responsible for the examination that can completely change decision from requesting clinician or surgeon. It is necessary to avoid a simply descriptive report, we must make considerations about differential diagnosis, suggest the approach to be adopted whenever possible, and, as I am used to say to my residents, "put name of the diseases on the reports".

Therefore, I would like to answer the question in the article's title: "Can high-resolution chest CT findings diagnose pulmonary alveolar microlithiasis?"... Yes, for sure!
